# γδ T Cells: Crosstalk Between Microbiota, Chronic Inflammation, and Colorectal Cancer

**DOI:** 10.3389/fimmu.2018.01483

**Published:** 2018-06-26

**Authors:** Yunben Yang, Chunjing Xu, Dang Wu, Zhen Wang, Pin Wu, Lili Li, Jian Huang, Fuming Qiu

**Affiliations:** ^1^Cancer Institute (Key Laboratory of Cancer Prevention and Intervention, National Ministry of Education), The Second Affiliated Hospital, Zhejiang University School of Medicine, Zhejiang University, Hangzhou, China; ^2^Department of Medical Oncology, The Second Affiliated Hospital, Zhejiang University School of Medicine, Zhejiang University, Hangzhou, China; ^3^Department of Surgical Oncology, The Second Affiliated Hospital, Zhejiang University School of Medicine, Zhejiang University, Hangzhou, China; ^4^Department of Radiation Oncology, The Second Affiliated Hospital, Zhejiang University School of Medicine, Zhejiang University, Hangzhou, China; ^5^Department of Thoracic Surgery, The Second Affiliated Hospital, Zhejiang University School of Medicine, Zhejiang University, Hangzhou, China

**Keywords:** γδ T cells, microbiota, chronic inflammation, colorectal cancer, tumor microenvironment

## Abstract

Increasing evidence suggests that intestinal microbiota dysbiosis and chronic inflammation contribute to colorectal cancer (CRC) development. γδ T cells represent a major innate immune cell population in the intestinal epithelium that is involved in the maintenance of gut homeostasis, inflammation regulation, and carcinogenesis. The important contributions of γδ T cells are (i) to perform a protective role in the context of barrier damage and pathogenic microorganism translocation; (ii) to exert either pro- or anti-inflammatory effects at different inflammatory stages; and (iii) to boost the crosstalk between immune cells and tumor microenvironment, inducing a cascade of suppressive immune responses. Understanding the crucial role of γδ T cells would enable us to manipulate these cells during the CRC sequence and improve the efficacy of tumor therapy.

## Introduction

Colorectal cancer (CRC) is the third most-common cancer in the world with high mortality (1–3). Evidence suggests that CRC development is linked to the change of environmental factors including dietary behavior, obesity, and infection, which further lead to the alteration of microbial composition (4, 5). The majority of the microbiota resides on the surface of the intestine and is indispensable for physiologic homeostasis; microbial dysbiosis has been linked to chronic inflammation and cancer development (6–8). Metagenomic analysis of specimens (mucosal tissues or fecal samples) from patients with CRC and from healthy controls shows dramatic differences in the microbial community structure (9–11). Moreover, compared to those colonized with healthy microbiota, transfer of cancer-associated microbiota into germ-free mice significantly increases the tumor burden, indicating that altered microbiota exacerbate CRC formation (12–15). From a mechanistic point of view, microbiota can generate potential oncometabolites that reshape the polarization of immune cells, thus contributing to aberrant inflammatory processes and loss of prevention of inappropriate immune responses (16–18). For example, reduced abundance of butyrate-producing bacteria, in patients with CRC, is associated with impaired barrier function and regulatory T cell generation (19). Dysbiosis-driven chronic inflammation evolves as a characteristic that fosters hallmarks of cancer, and triggers uncontrolled immune responses, resulting in loss of homeostasis and consequently in a vicious cycle contributing to tumorigenesis (16, 20). Altered commensal bacteria, chronic inflammation, and CRC development do not exist independently; they influence each other in reciprocal causation. Alterations of microbiota can occur prior to the initial macroscopic observation of colonic tumor formation, and bacterial translocation across the barrier may cause enhanced chronic inflammation (13); the recruitment of immune cells and changes in cancer cell metabolism drive the microbial structure remodeling (21). Innate immune cells act as significant mediators in these processes by participating in bidirectional regulation of inflammatory responses (22).

γδ T cells, a unique population of innate-like T lymphocytes, recognize the superstructure of antigen without the requirement of major histocompatibility complex (MHC) molecules (22, 23). In humans, the two major subsets of γδ T cells, comprising of Vδ1^+^ and Vγ9Vδ2 T cells, are identified by their Vδ chain usage. Vδ1^+^ (associated with various Vγ elements) T cells are the initial γδ T cells derived from thymus, which ultimately reside in colonic epithelial tissues and make up the majority of intraepithelial lymphocytes (IELs). Normal development and antimicrobial capacity acquisition of Vδ1^+^ T cells rely on each cell receiving proper instructions from the gut microbiota (24, 25). During microbial dysbiosis, resident Vδ1^+^ T cells are indispensable for triggering early protective inflammatory responses, which are essential for wound healing (22, 24, 26). Once the inflammatory response persists, Vδ1^+^ T cells subtly provide the foundation for angiogenesis, survival signal pathway activation, and myeloid-derived suppressor cell (MDSC) recruitment, thereby generating a chronic pre-cancerous inflammatory environment (27–29). In such cancer or stressed cells, isopentenyl pyrophosphate (IPP) gradually accumulates and boosts the activation of Vγ9Vδ2 T cells, which only take up a small fraction of lymphocytes in peripheral blood (PB), but harbor an antineoplastic effect (30, 31). However, the local tumor microenvironment (TME) has a profound influence on these recruited Vγ9Vδ2 T cells, which are gradually polarized into cancer-promoting phenotypes (e.g., Th17-like cells) (32). Taken together, tumor progression is facilitated by continuous presence of inflammatory cells and cytokines in the TME (21, 33).

This review highlights the plasticity of γδ T cells in different environments, thereby linking microbiota, inflammation, and CRC.

## Induction of γδ T Cell Activation and Polarization by Microbiota

**Figure 1:**
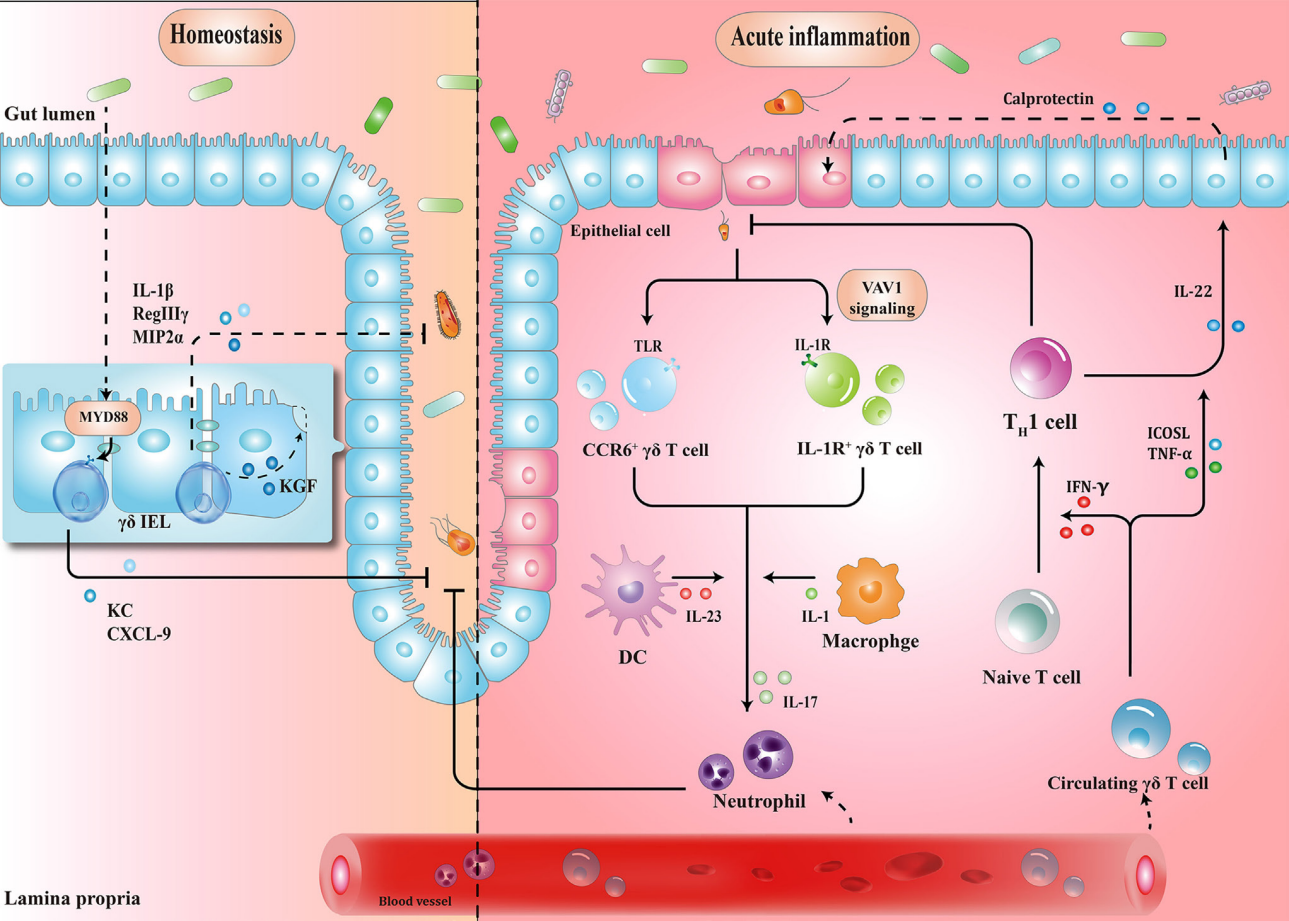
Schematic overview of the protective roles of γδ T cells in homeostasis maintenance and immune surveillance. (1) Physiologically, the crosstalk between microbiota, epithelial cells (ECs), and γδ T cells enhances barrier stabilization. (2) During acute inflammation, neutrophils are stimulated by IL-17 from γδ T cells, and recruited to eliminate pathogens. Meanwhile, microbe-activated circulating γδ T cells promote cytotoxic responses with Th1-committed αβ T cells and potentiate the release of calprotectin in an inducible T-cell co-stimulator ligand (ICOSL)/tumor necrosis factor α (TNF-α)-dependent manner. IEL, intraepithelial lymphocyte; MYD88, myeloid differentiation primary response 88; KC, keratinocyte-derived chemokine; CXCL, chemokine (C-X-C motif) ligand; MIP2α, macrophage inflammatory protein 2α; CCR, C-C motif chemokine receptor; TLR, toll-like receptor; IFN-γ, interferon γ; IL-17, interleukin-17.

γδ IELs, the major population of IELs, are localized in the intestinal surface, establishing the most intimate communication with microbiota (34, 35). In fact, commensal bacteria are necessary for the γδ IELs to release protective factors following mucosal damage, through both myeloid differentiation primary response 88 (MYD88)-dependent [e.g., keratinocyte-derived chemokine and chemokine (C-X-C motif) ligand 9 (CXCL9)] and MYD88-independent (e.g., macrophage inflammatory protein 2α, RegIIIγ) pathways (36, 37). Compared to that in wild-type mice, expression of antimicrobial cytokines is significantly reduced in germ-free mice, which may, in turn, be restored by transplanting microbiota harvested from conventionalized mice (37). Microbial effects on γδ IELs can be mediated by epithelial cells (ECs) (38–40). In a recent study, Hoytema van Konijnenburg et al. indicated that antimicrobial responses and movement dynamics of γδ IELs were impaired in mice with MYD88-deficient ECs. Interestingly, EC-mediated microbial sensing also controls metabolic switch of γδ IELs in response to infection (38). Thus, a cellular network of microbiota, ECs, and γδ IELs, in which microbiota-stimulated ECs regulate motility and energy metabolism of γδ IELs, is essential for the maintenance of homeostasis (Figure 1).

When barrier damage results in microfloral translocation, γδ T cells are indispensable for triggering early acute inflammatory responses against the penetration of microbiota across the impaired intestinal mucosa (34). For example, TCRγδ-deficient mice are more susceptible to harmful microbial cues and show early spread of pathogens to other organs (37). Importantly, a compelling feature seen in γδ T cells is their ability to secrete interleukin-17 (IL-17) in early pathogen invasion, which seems to benefit the host initially (41–43). Mechanistically, it is probable that γδ T cells recognize pathogen-associated molecular patterns through a series of toll-like receptors (TLRs), indicating the direct effect of bacterial products to drive the expansion of γδ T cells (44). For example, in response to pathogenic bacteria, C-C motif chemokine receptor 6 (CCR6)^+^ γδ T cells (predominately Vγ2) produce IL-17 through TLR1 and TLR2, which in turn form a positive feedback loop to recruit immune cells, thus launching the inflammatory response to eliminate pathogens before Th17 cells get activated (43). As such, commensal bacteria-induced IL-1R1^+^ γδ T cells provide the main source of IL-17 *via* the guanine nucleotide exchange factor VAV1 (45). Microbial metabolites or antigens may also exert indirect effects on γδ T cells. In response to altered micro-environmental cues, dendritic cells (DCs) can communicate with IL-17-producing γδ T (γδT17) cells *via* cell-to-cell contact or different cytokines, revealing a crosstalk between the immune system and microbiota (Figure 1) (46).

Circulating Vγ9Vδ2 T cells strategically migrate from blood to the infected intestine (47, 48). On one hand, γδ T cells promote Th1-committed αβ T cell responses *via* interferon γ (IFN-γ) production and enhance the secretion of inflammatory cytokines including tumor necrosis factor α (TNF-α) (49, 50). On the other hand, γδ T cells, employed as antigen-presenting cells, potentiate the secretion of IL-22 by CD4^+^ cells and release of calprotectin in an inducible T-cell co-stimulator ligand (ICOSL)/TNF-α-dependent manner (Figure 1) (51).

These studies suggest that dysbiosis can induce γδ T cell activation to trigger early protective inflammatory responses.

## Participation of γδ T Cells in Chronic Inflammation

γδ T cells are continuously activated by sustained exposure to bacterial metabolites, thereby leading to the exhaustion of protective γδ T cell subtypes and activation of chronic inflammation (5, 18). In this regard, one representative example is inflammatory bowel disease (IBD), including Crohn’s disease (CD) and ulcerative colitis (UC) (52–54). Although γδ T cells (especially Vδ1^+^) are reported to be significantly increased in the inflamed mucosa of patients with UC, the role of γδ T cells in human CD remains a matter of debate [reviewed in Ref. (55)], suggesting the discrepancy of immunological background between UC and CD (55, 56). Early studies, focused on the change in proportion of γδ T cells in CD (either in PB or tissue samples), reported diverse results (57–60), probably due to the limitation of small sample size and different γδ T cell subpopulations. For example, a recent study compared γδ T cell subsets in PB from 40 patients having CD with that from healthy controls, and the reduction extent of γδ T cell subsets was addressed, especially for CD8^+^ γδ T cells (61). The authors showed that deficiency of this γδ T cell subset could affect the immune responses to pathogens in patients with CD (62). Lately, Kadivar et al. found decreased levels of CD8αβ^+^ γδ T cells (predominately Vδ1^+^) in inflamed mucosa, associated with worse disease activity, whereas increased proportion of CD8αβ^+^ γδ T cells was observed in anti-TNF-α-treated patients with CD (63). Based on these results, γδ T cells cannot be simply recognized as one homogeneous population; however, further studies would be required to define the functions of different γδ T cell subtypes for interpreting the pathology of human IBD.

**Figure 2:**
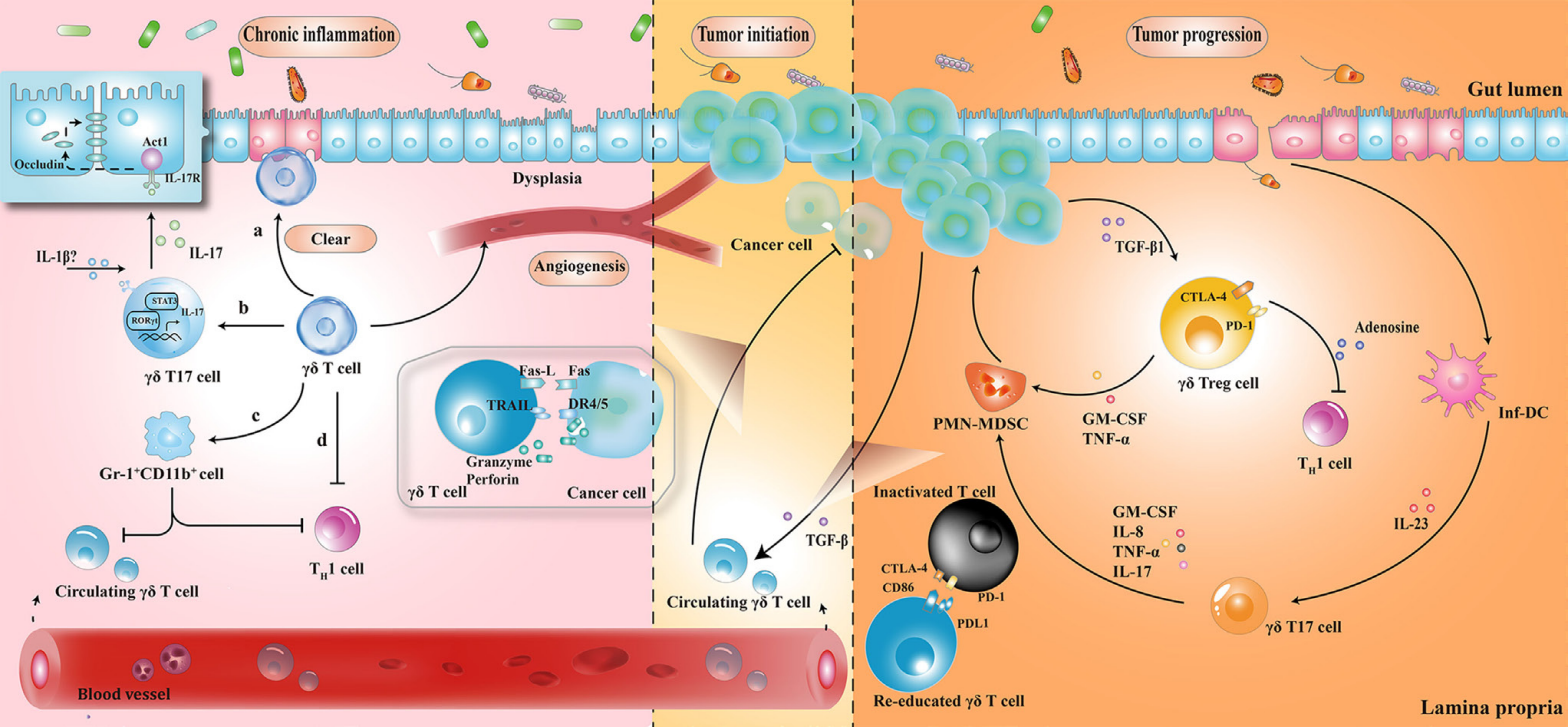
The roles played by γδ T cells at different stages of colorectal cancer development. (1) During chronic inflammation, γδ T cells can limit excessive inflammatory response and maintain homeostasis by (a) removal of impaired epithelial cells; (b) secretion of protective IL-17; (c) enhancement of Gr-1^+^CD11b^+^ suppressor cell infiltration; and (d) regulation of αβ T cell functions. (2) At the initiation of tumor formation, circulating γδ T cells can recognize and kill cancer cells, but they are reprogrammed with cancer development. (3) At stages of tumor progression, IL-17 and granulocyte macrophage colony-stimulating factor (GM-CSF), produced by γδ T cells in response to IL-23 and transforming growth factor beta 1 (TGF-β1) from inflammatory dendritic cells (inf-DCs) and cancer cells, are essential for the recruitment of polymorphonuclear myeloid-derived suppressor cells (PMN-MDSCs), consequently inducing a cascade of suppressive immune responses. CTLA4, cytotoxic T lymphocyte-associated protein-4; PD-1, programmed cell death protein 1; TNF-α, tumor necrosis factor α; IL-17, interleukin-17.

The protective role of γδ T cells in exerting wound-healing responses has been suggested in murine colitis model. γδ T cells preserve homeostasis by removal of impaired ECs; secretion of growth factors to promote epithelial regeneration; regulation of αβ T cell functions to limit excessive inflammatory response; and enhancement of granulocyte infiltration (64–66). Importantly, one layer of these processes is associated with protective IL-17 production (28, 41, 67); Lee et al. showed that γδ T cells were the major producers of protective IL-17 in the retinoid-related orphan receptor γt-dependent and IL-23-independent manner (68). Secretion of IL-17 could activate Act-1 (a key adaptor protein of IL-17 receptor), which attenuated inflammation and immobilized the localization of occluding (a tight junction protein) to prevent excessive intestinal permeability (Figure 2) (68). Another crucial anti-inflammatory pathway of γδ T cells is linked to the recruitment of MDSCs (29). Sun et al. observed that TCRγδ-deficient mice were more susceptible to dextran sulfate sodium-induced colitis, with a reduced expression of IL-18 and CXCL5 relative to wild-type mice, which was important for the subsequent Gr-1^+^CD11b^+^ suppressor cell infiltration (29). Moreover, mice reconstituted with γδT17 cells (mainly Vγ6) had ameliorated intestinal inflammation and were associated with increased frequency of Gr-1^+^CD11b^+^ cells, whereas mice with IFN-γ producing γδ T cells had no significant difference, indicating a protective role of γδT17 cells (but not IFN-γ producing γδ T cells) to a certain extent (29).

Taken together, γδ T cells seem to maintain tissue architecture during chronic inflammation, probably *via* IL-17 production and MDSC accumulation. However, it is noteworthy that these mediators have the ability to support tumor development by enhancing angiogenesis and promoting immunological tolerance to tumor cells. While these mechanisms have not been demonstrated in patients with IBD, the complex interactions between γδ T cells and MDSCs hint at the possible regulatory pathways in humans.

## The Facilitative Role of γδ T Cells in CRC

In tumor initiation, circulating Vγ9Vδ2 T cells, activated by the over-produced IPP in cancer cells, express the inflammatory homing chemokine receptors (e.g., CXCR3 and CCR5), guiding them into tumor sites (69, 70). By identifying the various upregulated ligands in cancer cells (e.g., MHC class I-related chains A/B and UL16-binding proteins), Vγ9Vδ2 T cells can either kill tumor cells indirectly by releasing abundant cytokines (e.g., IFN-γ and TNF-α), thereby displaying a Th1 cell-like property, or do so directly *via* death receptor signal (e.g., Fas-FasL and TNF-TNFR), secreting cytotoxic molecules such as granzymes (71, 72). By contrast, with tumor progression, tumor-associated macrophages and MDSCs are enriched in TME, acting as endless suppliers of IL-1β, IL-23, and transforming growth factor beta (TGF-β). Therefore, the recruited Vγ9Vδ2 T cells are reprogrammed by the local TME and may be polarized to γδT17 cells or regulatory γδ T cells (γδTregs). The polarized γδT17 cells or γδTregs produce abundant IL-17 or TGF-β, triggering a cascade of suppressive immune responses to promote CRC progression (Figure 2) (32, 73, 74).

The local TME has a considerable impact on tissue-infiltrating Vδ1^+^ T cells as well. Interestingly, γδ T cells were found to be the major source of IL-17 and majority of tumor-infiltrating γδT17 cells were Vδ1^+^ (21). Epithelial barrier damage was found to cause liberation of microbial products, resulting in activation of inflammatory DCs (inf-DCs), which polarized γδT17 cells in an IL-23-dependent manner (21). Tumor-infiltrating γδT17 cells could secrete IL-17, IL-8, TNF-α, and granulocyte macrophage colony-stimulating factor to promote the proliferation and survival of polymorphonuclear MDSCs, thus transforming the CRC-triggered inflammation into an immunosuppressive condition (21). In addition to indirect immunosuppressive role of γδT17 cells, a novel population of CD39^+^γδTregs was identified, which mediated a direct and robust immunosuppressive effect on human CRC (75). CD39^+^γδTregs took up nearly 50% of the total γδ T cells and expressed high levels of suppressive molecules (such as Forkhead box p3, cytotoxic T lymphocyte-associated protein-4, and programmed cell death protein 1), as well as cytokines (including IL-10 and IL-17), contributing to the formation of a potent immunosuppressive microenvironment. Stimulated by TGF-β1, CD39^+^γδTregs could secrete adenosine to foster CRC progression (Figure 2) (75). More importantly, the frequency of γδT17 and CD39^+^γδTregs was related to clinicopathological factors in patients with CRC (e.g., TNM stages, tumor size, and invasion), suggesting them to be potential prognostic factors (21, 75). Similarly, Rong et al. described a disproportionate distribution of Vδ1^+^ and Vδ2^+^ T cells in rectal cancer tissues (76). A higher percentage of Vδ1^+^ T cells and a lower percentage of Vδ2^+^ T cells were found in the tumor tissues compared to that in the adjacent normal tissues. Tumor-infiltrating Vδ1^+^ T cells had strong inhibitory functions and positively associated with the T stage of patients (76).

## Conclusion and Future Direction

γδ T cell is a key mediator of barrier surveillance, chronic inflammation regulation, and immunosuppressive TME formation. In this review, we focused on the dynamic changes of γδ T cell infiltration during the prolonged period of intestinal architectural disruption, suggesting that milieu alteration is a decisive factor for cell polarization. Based on recent reports, a potential mechanistic framework has been elucidated (Figures 1 and 2). In fact, we have just begun to uncover the implication of γδ T cells in the CRC sequence. For example, although utilization of γδ T cells (mainly Vγ9Vδ2) (adoptive transfer of *in vitro* expanded cells or *in vivo* activation) has been employed in a series of clinical trials, the clinical responses of CRC treatment are barely satisfactory (77–80). Several additional factors should be considered for future γδ T cell immunotherapy: primarily, the impact of local TME should be seriously considered. Despite transmutation of Vγ9Vδ2 T cells from anti-tumoral to pro-tumoral activity can occur in TME, the CRC-derived Vδ1^+^ tumor-infiltrating lymphocytes have been shown to kill colonic cancer cells *in vitro* (81–83). Therefore, it confirms that environmental changes can lead to remodeling of cell function. Moreover, the difference of microbial community structure between individuals, as well as the interaction between gut microbiota and γδ T cells is also worth considering. Questions such as: whether alteration of a certain species of microorganisms is the cause or consequence in the alteration of γδ T cell subsets, which microbiota-derived metabolites can be certainly related to the polarization of γδ T cells, what are the exact mechanisms resulting from microbiota dysbiosis or chronic inflammation that cause skewed responses of γδ T lymphocytes, whether pro-tumoral activities of γδ T cells benefit from commensal penetration, and in turn facilitate uncontrolled inflammation and tumor formation, or whether γδ T cells can be reprogrammed by precise regulation of the microbiota composition to avoid chronic inflammatory microenvironment transformation, will need to be addressed, for the manipulation of γδ T cell-based immunotherapy to improve the efficacy of CRC treatment.

## Author Contributions

Conception and design: FQ and JH. Write, review, and revision of the manuscript: YY, CX, DW, and ZW. Supervision: PW and LL.

## Conflict of Interest Statement

The authors declare that the research was conducted in the absence of any commercial or financial relationships that could be construed as a potential conflict of interest. The reviewer LW declared a shared affiliation, though no other collaboration, with the authors to the handling Editor.
